# Long-Term Exercise Reduces Formation of Tubular Aggregates and Promotes Maintenance of Ca^2+^ Entry Units in Aged Muscle

**DOI:** 10.3389/fphys.2020.601057

**Published:** 2021-01-05

**Authors:** Simona Boncompagni, Claudia Pecorai, Antonio Michelucci, Laura Pietrangelo, Feliciano Protasi

**Affiliations:** ^1^Center for Advanced Studies and Technology (CAST), University G. d’Annunzio (Ud’A) of Chieti-Pescara, Chieti, Italy; ^2^Department of Neuroscience, Imaging and Clinical Sciences (DNICS), University G. d’Annunzio (Ud’A) of Chieti-Pescara, Chieti, Italy; ^3^Department of Medicine and Aging Sciences (DMSI), University G. d’Annunzio (Ud’A) of Chieti-Pescara, Chieti, Italy

**Keywords:** sarcoplasmic reticulum, transverse tubule, excitation-contraction coupling, electron microscopy, store opereted calcium entry

## Abstract

Tubular aggregates (TAs) in skeletal muscle fibers are unusual accumulation of sarcoplasmic reticulum (SR) tubes that are found in different disorders including TA myopathy (TAM). TAM is a muscular disease characterized by muscle pain, cramping, and weakness that has been recently linked to mutations in *STIM1* and *ORAI1.* STIM1 and ORAI1 are the two main proteins mediating store-operated Ca^2+^ entry (SOCE), a mechanism activated by depletion of intracellular Ca^2+^ stores (e.g., SR) that allows recovery of Ca^2+^ from the extracellular space during repetitive muscle activity. We have recently shown that exercise triggers the formation of unique intracellular junctions between SR and transverse tubules named *Ca*^2+^ *entry units* (CEUs). CEUs promote colocalization of STIM1 with ORAI1 and improve muscle function in presence of external Ca^2+^. TAs virtually identical to those of TAM patients are also found in fast-twitch fibers of aging male mice. Here, we used a combination of electron and confocal microscopy, Western blotting, and *ex vivo* stimulation protocols (in presence or absence of external Ca^2+^) to evaluate the presence of TAs, STIM1-ORAI1 localization and expression and fatigue resistance of intact extensor digitorum longus (EDL) muscles in wild-type male adult (4-month-old) and aged (24-month-old) mice and in mice trained in wheel cages for 15 months (from 9 to 24 months of age). The results collected indicate that (i) aging causes STIM1 and ORAI1 to accumulate in TAs and (ii) long-term exercise significantly reduced formation of TAs. In addition, (iii) EDL muscles from aged mice exhibited a faster decay of contractile force than adult muscles, likely caused by their inability to refill intracellular Ca^2+^ stores, and (iv) exercise in wheel cages restored the capability of aged EDL muscles to use external Ca^2+^ by promoting maintenance of CEUs. In conclusion, exercise prevented improper accumulation of STIM1 and ORAI1 in TAs during aging, maintaining the capability of aged muscle to refill intracellular Ca^2+^ stores *via* SOCE.

## Introduction

The sarcoplasmic reticulum (SR) is a highly organized system of membranes that functions as the main intracellular calcium (Ca^2+^) storage of skeletal muscle (Franzini-Armstrong, 1980; Boncompagni et al., 2020). In adult muscle fibers, the SR is composed of two distinct compartments in direct continuity with each other: the SR terminal cisternae or junctional SR (jSR) and the longitudinal SR (lSR) (Franzini-Armstrong, 1984). Among other few important proteins, the jSR contains ryanodine receptors (RYRs), large proteins that constitute Ca^2+^ release channels (Franzini-Armstrong and Protasi, 1997) and calsequestrin (CASQ), a protein that accumulates Ca^2+^ in proximity of release sites (Saito et al., 1984; Franzini-Armstrong et al., 1987). The jSR is tightly associated with transverse tubules (TTs) to form triads, also known as Ca^2+^ release units (CRUs). In adult mammalian skeletal muscle fibers, CRUs are placed in proximity of the A-I band transition of relaxed sarcomeres and contain the macromolecular complex that mediates excitation–contraction (EC) coupling (Schneider and Chandler, 1973; Schneider, 1994; Franzini-Armstrong and Protasi, 1997). EC coupling is the mechanism that translates the action potential carried in the fiber interior by TTs into Ca^2+^ release through RYRs. On the other hand, the lSR membranes are enriched in sarco/endoplasmic reticulum Ca^2+^ ATPases (SERCAs), which rapidly remove cytosolic Ca^2+^ released by RYRs during EC coupling to replenish SR lumen after each contraction (Inui and Fleischer, 1988). lSR extends on both sides of the terminal cisternae and thus may be placed either next to A or to I bands.

Tubular aggregates (TAs) are abnormal and extensive accumulation of ordered and tightly packed SR tubes, first described in 1970 by Engel and colleagues in muscle biopsies from dyskalemic patients (Engel et al., 1970). TAs are also found in other human muscle disorders, such as periodic paralysis or myotonic disorders (De Groot and Arts, 1982; Pierobon-Bormioli et al., 1985; Rosenberg et al., 1985; Morgan-Hughes, 1998; Vissing et al., 1999) and constitute a constant histopathological feature in TA myopathy (TAM), a relatively rare disorder linked to gain-of-function mutations in both the stromal-interacting molecule-1 (STIM1) SR Ca^2+^ sensor and the ORAI1 Ca^2+^ release–activated Ca^2+^ channel of the plasma membrane (Bohm et al., 2013, 2014, 2017; Nesin et al., 2014; Endo et al., 2015; Walter et al., 2015; Lee and Noguchi, 2016; Okuma et al., 2016).

First discovered in non-excitable cells, STIM1 and ORAI1 are the two main players that coordinate store-operated Ca^2+^ entry (SOCE) (Liou et al., 2005; Roos et al., 2005; Feske et al., 2006; Vig et al., 2006). SOCE is a ubiquitous Ca^2+^ influx mechanism triggered by reduction of Ca^2+^ levels in the lumen of intracellular Ca^2+^ stores (Putney, 1986, 2011a,b; Parekh et al., 1997). Also expressed in skeletal muscle (Kurebayashi and Ogawa, 2001; Lyfenko and Dirksen, 2008), STIM1/ORAI1-dependent SOCE is important in limiting fatigue during repetitive high-frequency stimulation (Zhao et al., 2005; Wei-Lapierre et al., 2013; Boncompagni et al., 2017; Michelucci et al., 2019 and 2020). Altered SOCE has been associated to muscle dysfunction in various myopathies (Pan et al., 2014; Michelucci et al., 2018). Importantly, it has been reported that a reduction in SOCE activity contributes to altered muscle function during aging (Zhao et al., 2008; Brotto, 2011; Thornton et al., 2011).

Under resting conditions in muscle, ORAI1 is located within the TT system, whereas STIM1 is mainly positioned throughout the lSR at the I band region of the sarcomere (Wei-Lapierre et al., 2013; Carrell et al., 2016; Boncompagni et al., 2017). We have recently shown that, in extensor digitorum longus (EDL) muscle fibers from adult wild-type (WT) mice, acute exercise drives the formation of Ca^2+^ entry units (CEUs), new intracellular junctions between stacks of lSR membranes, and TT extensions within the I band that contain colocalized STIM1 and ORAI1 (Boncompagni et al., 2017, 2018; Protasi et al., 2020). Interestingly, CEUs are constitutively assembled in fibers of mice lacking CASQ1, which undergoes severe Ca^2+^ depletion during repetitive high-frequency stimulation (Michelucci et al., 2020). The presence of these junctions correlates to (a) enhanced resistance to fatigue in presence of extracellular Ca^2+^ (Boncompagni et al., 2017) and (b) increased Ca^2+^ influx *via* SOCE (Michelucci et al., 2019, 2020).

Besides being found in muscle disorders, TAs have been also described during aging in EDL muscles from male mice, where they preferentially assemble in fast-twitch fibers (Salviati et al., 1985; Chevessier et al., 2005; Boncompagni et al., 2012). To our knowledge, presence of TAs has not been confirmed in aged human muscles. Consistently with their SR origin, TA tubes stain negatively for mitochondrial proteins while positively for proteins resident in SR membranes such as CASQ1 and SERCA. Nevertheless, the interior of the aggregate does not contain RYRs (Salviati et al., 1985; Chevessier et al., 2005; Boncompagni et al., 2012). Schiaffino and collegues showed that formation of aggregates is induced by anoxia in isolated rat muscle (Schiaffino et al., 1977).

We have shown that inactive aging impairs structure, function, and architecture of EC coupling and metabolic (i.e., mitochondria) machineries in muscle of mice and human biopsies (Boncompagni et al., 2006; Pietrangelo et al., 2015), changes that were reduced/counteracted by regular/lifelong exercise, which successfully prevented improper remodeling of intracellular membranes (Zampieri et al., 2015; Pietrangelo et al., 2019). Lifelong training combined to selenium supplementation were recently shown to reduce the age-related loss of muscle force and to improve Ca^2+^ release from RYR1 (Fodor et al., 2020).

In the current study, we analyzed EDL muscles from adult (4-month-old), aged controls (24-month-old), and aged trained mice (24-month-old mice housed in wheel cages for a period of 15 months starting from the age of 9 months), using a combination of structural (electron and confocal microscopy) and functional (*ex vivo* muscle contractility) approaches to test the effect of prolonged voluntary exercise on the accrual of TAs in muscle of aging mice.

## Materials and Methods

### Animals

All procedures and experiments in this study were conducted according to the National Committee for the protection of animals used for scientific purposes (D. lgs n.26/2014). C57bl/6 WT animals were housed in microisolator cages at 20°C in a 12 h light–dark cycle and provided free access to standard chow and water. All surgeries were made to minimize animal suffering, and animals were euthanized by cervical dislocation as approved by the D. lgs n.26/2014.

In this study, we compared three groups of male WT mice: (a) adult mice, mice of 4–6 months of age (*n* = 8); (b) aged control mice, mice of 24 months of age housed in regular cages (*n* = 10); and (c) aged trained mice, mice of 24 months of age housed from 9 to 24 months of age in wheel cages (*n* = 10). All mice had free access to standard chow and water. Aged trained mice were housed for 15 months in wheel cages for voluntary running (16 Station Home Cage Running Wheel System with CMI Software, Columbus Instruments, Columbus, OH, United States) starting at 9 months of age. Voluntary running activity was monitored in all cages with a sensor connected to a personal computer. In this study, we included only mice that ran a distance of at least 30 km per month from 9 to 20 months of age. At 24 months of age, mice were euthanized by cervical dislocation and processed for different preparations.

Only for data in Figure 2, WT mice of 4 months of age (*n* = 3) were exposed to a single bout of exercise protocol using a running treadmill (Columbus Instruments) as described in Boncompagni et al. (2017). Briefly, a first step of warm-up at low speed (10 min at 5 m/min) was used to familiarize the mice with the apparatus and task. The experimental exercise protocol started immediately after the warm-up session and was designed as follows: at the beginning of the protocol, the speed was set to 10 m/min for 25 min, then to 15 m/min for 20 min, then to 20 m/min for 15 min, and finally the speed was increased for 1 m/min every 1 min until the final speed of 25 m/min was reached (and kept for maximum 1 min). Mice were then euthanized and processed for electron microscopy.

### Preparation and Analysis of Samples for Histology and Electron Microscopy

Extensor digitorum longus muscles were quickly dissected from euthanized mice, pinned on a Sylgard dish, fixed at room temperature (RT) with 3.5% glutaraldehyde in 0.1 M NaCaCO buffer (pH 7.2), and stored in the fixative at 4°C before embedding. Fixed muscles were then postfixed, embedded, and stained *en bloc*, as described previously (Pietrangelo et al., 2015, 2019). For TT staining, specimens were postfixed in a mixture of 2% OsO_4_ and 0.8% ferrocyanide [K_3_Fe(CN)_6_] for 1–2 h followed by a rinse with 0.1 M NaCaCO buffer with 75 mM CaCl_2_. For histological examination, 700-nm-thick sections were stained in a solution containing 1% toluidine blue-O and 1% sodium borate (tetra) in distilled water for 3 min on a hot plate at 55–60°C. After washing and drying, sections were mounted with mounting medium DPX Mountant for histology (Sigma–Aldrich, Milan, Italy) and observed with a Leica DMLB light microscope connected to a Leica DFC450 camera equipped with Leica Application Suite v 4.6 for Windows (Leica Microsystem, Vienna, Austria). For electron microscopy (EM), ultrathin sections (∼50 nm) were cut using a Leica Ultracut R microtome (Leica Microsystem, Vienna, Austria) with a Diatome diamond knife (Diatome, Biel, Switzerland) and double-stained with uranyl acetate replacement and lead citrate. Sections were viewed in an FP 505 Morgagni Series 268D electron microscope (FEI Company, Brno, Czechia), equipped with Megaview III digital camera and Soft Imaging System at 60 kV (Olympus Soft Imaging Solutions, Munster, Germany).

### Immunofluorescence Labeling and Confocal Microscopy

Extensor digitorum longus muscles were dissected from euthanized animals, fixed with 2% paraformaldehyde in phosphate-buffered saline (PBS) for 20 min at RT, and stored at 4°C overnight. Small bundles of fixed EDL fibers were washed three times in PBS containing 1% [PBS/bovine serum albumin (BSA)] and incubated in blocking solution (PBS/BSA with 10% goat serum and 0.5% Triton X-100) for 1 h at RT, followed by an overnight incubation at 4°C with one of the following primary antibodies: (a) mouse monoclonal anti-RYR1 (34C antibody, 1:30, Developmental Studies Hybridoma Bank University of Iowa, Iowa City, Iowa); (b) rabbit polyclonal anti-STIM1 (1:100, Sigma–Aldrich, Milan, Italy); and (c) rabbit polyclonal anti-ORAI1 (1:20, Thermo Fisher Scientific, Waltham, MA, United States). Bundles of EDL muscles were then incubated for 1 h at RT with the following secondary antibodies (Jackson ImmunoResearch Laboratories, Lexington, KY, United States): Cy5-labeled goat anti–mouse immunoglobulin G (IgG) (1:100); and Cy3-labeled goat anti–rabbit IgG (1:200) for double labeling. Confocal images were acquired using a Zeiss LSM510 META laser-scanning confocal microscope system (Zeiss, Jena, Germany) equipped with a Zeiss Axiovert 200 inverted microscope and a Plan Neofluar oil-immersion objective (100 × /1.3 NA).

### Quantitative Analysis in Histology and EM

For quantitative histological analyses, images of non-overlapping regions were randomly collected from transversal sections of internal areas of EDL fibers from adult (*n* = 3), aged (*n* = 3), and aged trained (*n* = 3) mice.

We evaluated the percentage of fibers containing TAs, the number of TAs per fiber, and the average size of TAs.

For quantitative EM analyses, micrographs of non-overlapping regions were randomly collected from transversal sections of internal areas of EDL fibers from adult (*n* = 3), aged (*n* = 3), and aged trained (*n* = 3) mice.

(1) The number of stacks in 100 μm^2^ of EM section was determined from electron micrographs at 28,000 × magnification. In each fiber, five micrographs were taken.
(2) The extension of non-triadic TT network at the I band (TT length in microns) per 100 μm^2^ of cross-sectional area was measured in electron micrographs at 28,000 × magnification and reported as length (micron)/100 μm^2^. In each fiber, five micrographs were taken.
(3) The junctional gap covered by electron dense linkers between SR vesicles in adult control muscle fibers and SR stacks in adult exercised mice was measured as previously reported (Boncompagni et al., 2017). Linkers between tubes of TAs in aged muscle were measured in electron micrographs at 56,000 × magnification; sample size: three mice, nine micrographs, and 50 measurements.

### *Ex vivo* Fatigue Protocol

*Ex vivo* assessment of muscle force production during repetitive high-frequency stimulation was made in intact EDL muscles of adult (*n* = 5), aged (*n* = 6), and aged trained (*n* = 6) mice. Briefly, muscles were excised from hind limbs, placed in a dish containing a standard Krebs–Henseleit (KH) solution: (118 mM NaCl, 5 mM KCl, 2.5 mM CaCl_2_, 1 mM KH_2_PO_4_, 1 mM MgSO_4_, 25 mM NaHCO_3_, and 11 mM glucose; pH 7.4) pinned and tied with fine silk sutures at each end. Muscles were then mounted vertically between two platinum electrodes immersed in an organ chamber filled with KH solution and attached to a servo motor and force transducer (model 1200 A; Aurora Scientific, Aurora, ON, Canada). Before starting the experimental protocol, stimulation level and optimal muscle length (L_0_) were determined using a series of 80-Hz stimulus strains every 1 min in order to adjust the muscle to the length that generated maximal force (F_0_) and avoid muscle fatigue. Twitch and tetanic contractile properties were then measured. Following these baseline measurements, EDL muscles were subjected to a repetitive high-frequency stimulation fatigue protocol consisting of 30 consecutive, 1 s duration, 60-Hz stimulus trains delivered every 5 s while being continuously perfused with KH solution. To assess the relative contribution of extracellular Ca^2+^ entry, other experiments were conducted under conditions designed to limit/block Ca^2+^ entry, including (i) nominally Ca^2+^-free KH solution (where external Ca^2+^ was replaced with an equimolar amount of Mg^2+^) and (ii) standard KH solution supplemented with 10 μM BTP-2, an established inhibitor of SOCE (Zitt et al., 2004). Before starting the repetitive high-frequency stimulation protocol, EDL muscles were equilibrated in either Ca^2+^-free KH solution or standard KH solution plus BTP-2 for a period of at least 20 min. Muscle force was recorded using Dynamic Muscle Control software and analyzed using a combination of Dynamic Muscle Analysis (Aurora Scientific) software. Specific force (mN/mm^2^) was calculated by normalizing the absolute force (mN) to the physiological cross-sectional area (mm^2^) obtained as follows: wet weight (mg)/[L_0_ (mm) × 1.06 (mg/mm^3^) × 0.44] (Hakim et al., 2011; Michelucci et al., 2019, 2020). All experiments were carried out at RT.

### Western Blot Analyses

Extensor digitorum longus muscles were dissected from adult (*n* = 4), aged (*n* = 4), and aged trained (*n* = 4) mice and homogenized in a lysing buffer containing 3% sodium dodecyl sulfate (Sigma–Aldrich, Milan, Italy) and 1 mM EGTA (Sigma–Aldrich, Milan, Italy) using a mechanical homogenizer and then centrifuged for 15 min at 900 × *g*, at RT. Protein concentration was determined spectrophotometrically using a modified Lowry method. Total protein (20–40 μg) was resolved in 10% polyacrylamide electrophoresis gels, transferred to nitrocellulose membrane, and blocked with 10% non-fat dry milk (EuroClone, Milan, Italy) in Tris-buffered saline and 0.1% Tween 20 (TBS-T) for 1 h. Membranes were then probed with primary antibodies diluted in 10% non-fat dry milk in TBS-T overnight, at 4°C: (a) rabbit polyclonal anti-STIM1 (1:5,000, Sigma–Aldrich, Milan, Italy); (b) rabbit polyclonal anti-ORAI1 (1:1,000, Santa Cruz Biotechnology, Dallas, TX, United States). The anti–glyceraldehyde-3-phosphate dehydrogenase antibody (mouse monoclonal, 1:15,000; OriGene Technologies, Rockville, MD, United States) was used as a loading control. Membranes were then incubated for 1 h at RT with mouse and rabbit secondary horseradish peroxidase–conjugated antibodies (1:10,000; Merck Millipore, Darmstadt, Germany), diluted in 10% non-fat dry milk in TBS-T. Proteins were detected by enhanced chemiluminescent liquid (Perkin-Elmer, Milan, Italy) and quantified using ImageJ software (National Institutes of Health, Bethesda, MD, United States).

### Statistical Analyses

Statistical significance was determined using either two-tailed unpaired Student *t* test when comparing means between two groups or one-way analysis of variance (ANOVA) followed by *post hoc* Tukey test when comparing more than two groups. For *ex vivo* contractile experiments, significance was evaluated using repeated-measurements ANOVA followed by *post hoc* Tukey test. All data were presented as mean ± SEM. In all cases, differences were considered statistically significant at ^∗^*p* < 0.05 (or ^∗^*p* < 0.01 where indicated).

## Results

### TAs Are STIM1 and ORAI1 Positive

**FIGURE 1:**
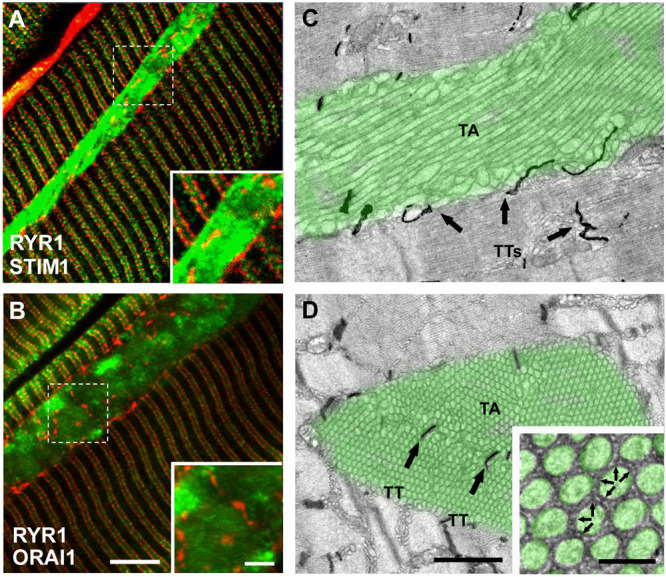
Immunofluorescence and EM analysis of EDL fibers from aged-control mice. **(A,B)** Representative immunofluorescence images obtained from aged mice, double-labeled for RYR1 (red) and STIM1 (green) in panel **(A)** and RYR1 (red) and ORAI1 in panel **(B)**. Raw images for the individual fluorescence channel used to construct these overlays are shown in Supplementary Figure 1. **(C,D)** Representative EM images of longitudinal **(C)** and transversal **(D)** sections with TAs false-labeled in green (arrows point to TTs stained with ferrocyanide) and TTs stained with ferrocyanide (dark precipitate). Black arrows in panel **(C,D)** point to TTs within the interior of the TA. Inset in panel **(D)** small bridges, pointed by small arrows, are visible between membranes of adjacent cross-sectioned tubes. Scale bars: **(A,B)**, 5 μm (insets 2 μm); **(C,D)**, 1 μm (inset 0.1 μm).

To evaluate the presence of STIM1 and ORAI1 within TAs, we immunostained EDL muscles from aged and aged trained mice with antibodies for STIM1 and ORAI1 (Figure 1 and Supplementary Figure 1). Fibers were double-labeled for RYR1 and STIM1 (Figure 1A) and for RYR1 and ORAI1 (Figure 1B): RYR1 staining (used to mark the position of CRUs/triads) produced the typical transverse cross striation corresponding to the position of CRUs at the A–I band junction, on both sides of Z-lines (Figures 1A,B; red staining). TAs in immunofluorescence images appear as elongated, spindle-shaped regions strongly positive to both STIM1 and ORAI1 antibodies (Figures 1A,B and Supplementary Figures 1B,E; green staining), whereas RYR1 staining is mainly excluded from the core of TAs, even if some positive spots of RYR1 are present.

STIM1 staining in TAs appears always very dense and quite uniform, whereas ORAI1 staining pattern was apparently less dense and more patchy (Figures 1A,B; insets) (see also Supplementary Figure 1). Note that the staining of STIM1 and ORAI1 within the aggregate did not overlap with the staining of RYR1 (marking the position of CRUs, which also contain TTs). As ORAI1 is a Ca^2+^-permeable channel of the plasma membrane located in TTs in skeletal muscle (Wei-Lapierre et al., 2013; Boncompagni et al., 2017), the lack of colocalization with RYR1 suggests that part of ORAI1 may be trapped inside the SR membranes of TAs.

The presence of some RYR1-positive spots in TAs reflects the presence of few CRUs/triads at the interface between different SR domains that constitute large TAs. This was confirmed by ferrocyanide staining in EM, a technique that creates a dark precipitate inside the lumen of TTs (Figures 1C,D): TTs are mainly excluded from the aggregate core and confined at the edge of each TA (dark precipitate in Figure 1C). However, TTs were sometimes trapped between multiple smaller aggregates that fuse to form larger one (Figure 1D; arrows) [see Boncompagni et al. (2012) for additional detail].

**FIGURE 2:**
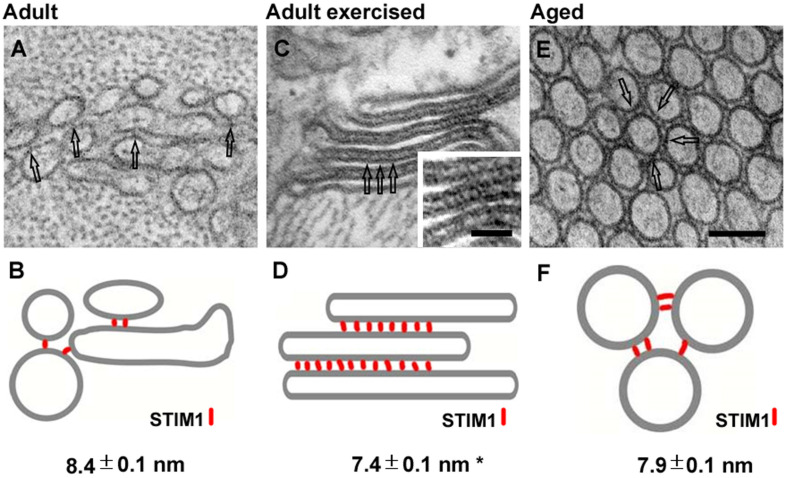
Representative EM images and corresponding cartoons showing SR linkers. SR vesicles in adult control mice [**(A)**, and relative cartoon in panel **(B)**], SR stacks in adult exercised mice [**(C)**, and relative cartoon in panel **(D)**], and TA tubes in aged EDL muscle fibers [**(E)**, and relative cartoon in panel **(F)**]. SR linkers are pointed by empty arrows in EM images and represented as red rods in the cartoons. Image and numeric data in panels **(B,D)** originates from adult mice acutely exercised in Boncompagni et al. (2017). Data are shown as mean ± SEM; *p < 0.01 (adult vs. adult exercised). Scale bar: **(A,C,E)**, 0.1 μm; inset, 0.05 μm.

Observation of TAs at high magnification revealed the presence of small electron-dense bridges between individual tubes (Figure 1D; arrows in inset). In Figure 2, we measured the length of these little bridges (that apparently keep the individual SR tubes together): their average size of 7.9 ± 0.1 nm is quite similar to that (i) of small linkers that are present between SR vesicles/tubes at the I band in adult control mice (8.4 ± 0.1 nm) and (ii) of linkers seen in SR stack of membranes forming CEUs that assemble during acute exercise in adult mice (7.4 ± 0.1 nm) (Figure 2) [see also Boncompagni et al. (2012, 2017)].

### Exercise Prevents Formation of TAs

**FIGURE 3:**
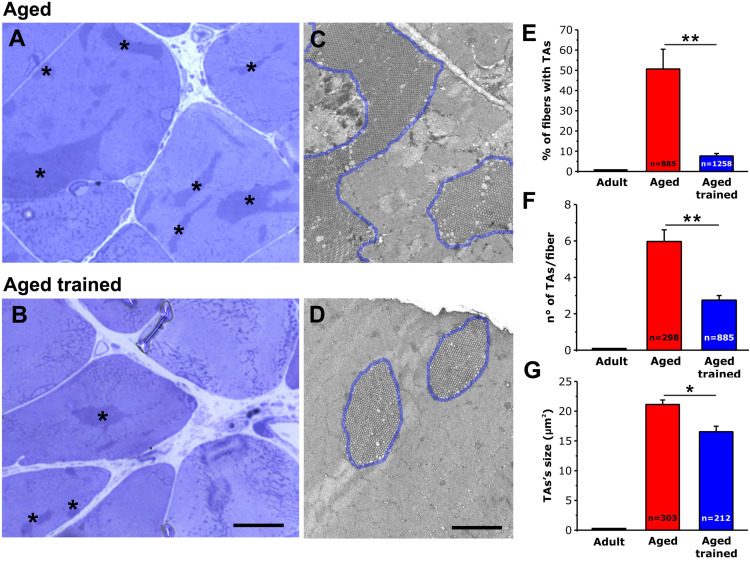
Quantitative histological and EM analysis of TAs incidence in EDL fibers. **(A–D)** Representative histological **(A,B)** and EM **(C,D)** images from transversal sections of EDL muscles from aged **(A,C)** and aged trained **(B,D)** mice. TAs are marked with an asterisk in panels **(A,B)**, whereas TAs are outlined with a blue line in panels **(C,D)**. Representative histological and EM images of adult EDL muscle (which do not contain TAs) are shown in Supplementary Figure 2. **(E–G)** Bar plots showing the quantitative analysis of the percentage of EDL fibers containing TAs **(E)**, the number of TAs per fiber **(F)**, and finally the average size of TAs **(G)**. Data are shown as mean ± SEM; **p < 0.01 in panels **(E,F)**; *p < 0.05 in G; *n* = number of fibers analyzed. Scale bars: **(A,B)**, 10 μm; **(C,D)**, 2 μm.

Using histological images taken from transversal sections of EDL muscles from aged and aged trained mice (Figures 3A,B), we evaluated: (i) the percentage of fibers presenting TAs (Figure 3E); (ii) the number of TAs per fiber (Figure 3F); and (iii) the average size of TAs (Figure 3G). As a visual example of TAs size, TA profiles were outlined in blue in the EM cross-sectional images of Figures 3C,D. We also performed similar analyses in muscles from adult mice, which do not contain TAs (Figures 3E–G and Supplementary Figure 2). Quantitative analysis of the percentage of fibers containing TAs indicated that exercise was quite effective in preventing their formation during aging. Specifically, the number of fibers containing TAs was reduced from ∼51% to only ∼8% following voluntary wheel running (Figure 3E). In addition, voluntary exercise was also effective in reducing (i) the average number of TAs per fiber from 6 to 3 (Figure 3F) and (ii) the average size of the few remaining TAs from 21 to 17 μm^2^ (Figure 3G) (see also Supplementary Table 1).

### Exercise Restores Resistance to Fatigue and Extracellular Ca^2+^ Dependence of EDL Muscles From Aged Mice

**FIGURE 4:**
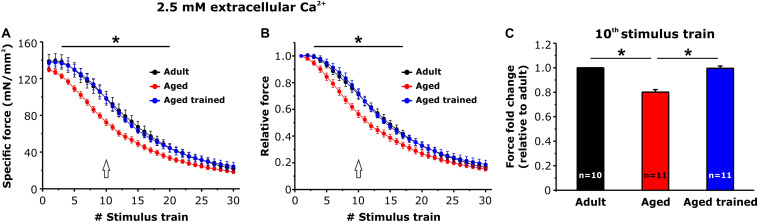
*Ex vivo* fatigue protocols in EDL muscles. **(A,B)** Time course of specific **(A)** and relative **(B)** force decay (normalized to the first stimulus train) during 30 consecutive stimulus trains (60 Hz, 1-s duration, every 5 s) in presence of a standard KH solution containing 2.5 mM Ca^2+^. The asterisks in panels **(A,B)** indicate the window in which there is a significant statistical difference between aged (control) and the other two groups of samples (adult and aged trained). **(C)** Bar plot showing the fold change of force, relative to adult mice, calculated at the 10th stimulus train [pointed by arrows in panels **(A,B)**]. Data are shown as mean ± SEM; *p < 0.05; *n* = number of EDL muscles.

**FIGURE 5:**
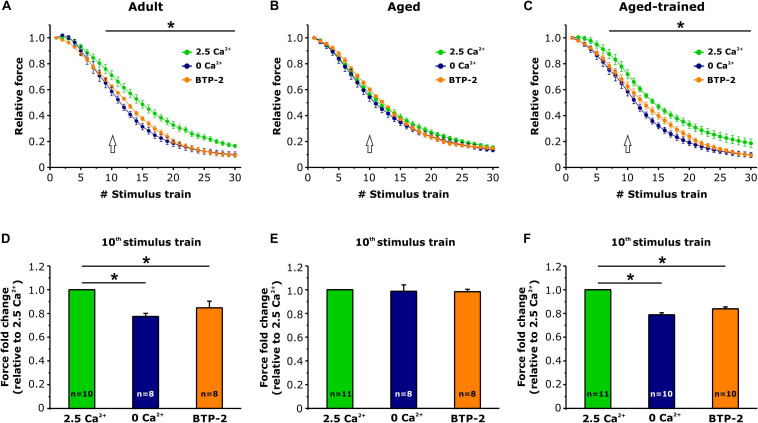
*Ex vivo* fatigue protocols in EDL muscles in presence or absence of extracellular Ca^2+^. **(A–C)** Time courses of relative force decay (normalized to the first stimulus train) during 30 consecutive stimuli (60 Hz, 1-s duration, every 5 s) in presence of standard KH solution either containing 2.5 mM Ca^2+^ (green), nominally Ca^2+^-free solution (dark blue), or standard KH solution containing 2.5 mM Ca^2+^ and supplemented with 10 μM BTP-2 (orange). The asterisks in panels **(A,C)** indicate the window in which there is a significant statistical difference between presence of 2.5 mM Ca^2+^ and the other two conditions (0 Ca^2+^ and presence of BTP2). **(D–F)** Bar plots showing the fold change of the force, relative to the 2.5 mM Ca^2+^ condition, calculated at the 10th stimulus train [see arrows in panels **(A–C)**]. Data are shown as mean ± SEM; *p < 0.05; *n* = number of EDL muscles.

Intact EDL muscles dissected from adult, aged, and aged trained mice were subjected to fatigue protocols based on 30 consecutive 1-s-long, 60-Hz stimulus trains applied every 5 s. The experiments were carried out using a standard KH solution containing 2.5 mM Ca^2+^ (Figure 4) or under conditions designed to abolish Ca^2+^ entry, e.g., nominally Ca^2+^-free KH solution or standard KH solution supplemented with 10 μM BTP-2 (Figure 5). In presence of the standard KH solution containing 2.5 mM Ca^2+^, EDL muscles from aged mice exhibited both a reduced specific force during the first stimulus train and a more pronounced drop in either specific and relative force generation during repetitive high-frequency stimulation (e.g., accelerated fatigue) compared to that observed in muscles from adult mice (Figures 4A,B). The quantitative analysis of the force fold change relative to the force generated by muscles from adult mice (Figure 4C), evaluated at the 10th stimulus train (pointed by arrows in Figures 4A,B), indicated that the decrease of force in muscles from aged mice was about 20%. Interestingly, following 15 months of voluntary wheel running, EDL muscles from aged trained mice exhibited a completely recovered ability to maintain contractile force (Figures 4A,B), as the average force fold change displayed by muscles from aged trained mice was not significantly different from that of adult muscles (Figure 4C). Parallel measurements were also performed under conditions designed to limit/block Ca^2+^ entry (Figure 5): (a) in a first set of experiments, muscles were exposed to a nominally Ca^2+^-free KH solution, where Ca^2+^ was replaced by an equimolar concentration of Mg^2+^; (b) in a second set of experiments, muscles were exposed to a standard KH solution supplemented with 10 μM BTP-2. Consistent with previous studies (Boncompagni et al., 2017; Michelucci et al., 2019), these interventions exhibited a modest, but statistically significant, effect on force production during repetitive stimulation in EDL muscles from adult mice (Figure 5A), although in the absence of Ca^2+^ entry (e.g., when external Ca^2+^ is removed or in the presence of BTP-2) EDL muscles from aged mice did not show any significant reduction of force generation compared to the standard condition (Figure 5B), suggesting their impaired capability to use external Ca^2+^. On the other hand, similarly to what was observed in muscles from adult mice, muscles from aged trained mice displayed a modest, but statistically significant, reduction in contractile force when Ca^2+^ influx was prevented (Figure 5C). The quantitative analysis of the fold force decay evaluated at the 10th stimulus train (pointed by arrows in Figures 5A–C) revealed that, in the absence of Ca^2+^ entry, EDL muscles from adult and aged trained mice exhibited a reduction in force of 15–20% compared to the condition in which Ca^2+^ entry was permitted (Figures 5D,F). Conversely, no significant difference of contractile force was reported in muscles of aged mice, in presence or absence of external Ca^2+^ (Figure 5E).

### Exercise-Induced Remodeling of SR and TT Membranes (i.e., CEU Components) in Aged Trained Muscles

**FIGURE 6:**
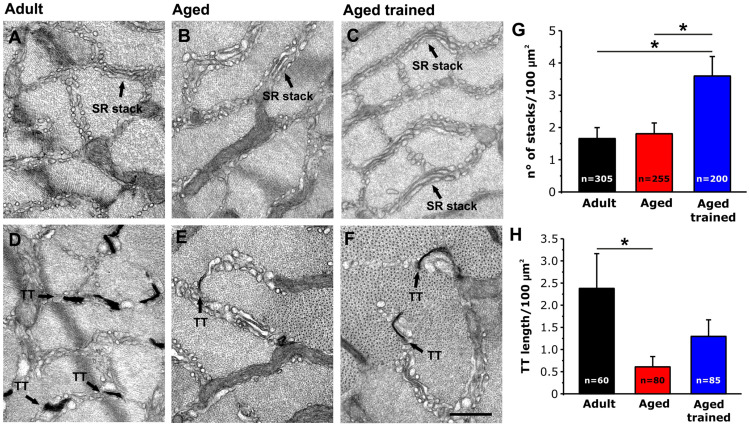
Quantitative analysis by EM of SR stacks and TT length at the I band in EDL fibers. **(A–F)** Representative EM images of transversal sections of EDL fibers from adult **(A,D)**, aged **(B,E)** and aged trained **(C,F)** mice. In **(D–F)** panels, TTs are stained with ferrocyanide (dark precipitate). **(G,H)** Bar plots showing the incidence of SR stacks **(G)** and the TT length at the I band **(H)**. Arrows point to SR stacks in panels **(A–C)**; arrows point to TTs stained with ferrocyanide in panels **(D–F)**. Data are shown as mean ± SEM; *p < 0.05; *n* = number of measurements. Scale bar: **(A–F)**, 0.1 mm.

As EDL muscles from aged trained mice exhibited a recovered capability to use external Ca^2+^ during repetitive stimulation (Figures 4, 5), we assessed and quantified the presence of the structural components needed for the assembly of Ca^2+^ entry units (CEUs): SR stacks (Figures 6A–C) and TT extension at the I band (Figures 6D–F).

Using EM, we quantified the number of SR stacks per area of cross section (Figure 6G) and the extent of the TT network at the I band following staining with ferrocyanide (Figure 6H). This analysis revealed that long-term voluntary running promoted (i) the formation of SR stacks in EDL muscles from aged trained mice, which were more numerous than those observed in muscles from both adult and aged mice (Figure 6G); (ii) elongation of TT at the I band, which is increased compared to aged mice, but not rescued to the levels of adult mice (Figure 6H) (see also Supplementary Table 2). As we previously showed that STIM1 and ORAI1 colocalize at SR-TT junctions formed by SR stacks and TT at the band (Boncompagni et al., 2017); here, we assessed the expression levels of the two proteins by performing Western blot experiments in EDL muscle homogenates (Supplementary Figure 3). The results of these experiments showed that expression of STIM1S and ORAI1 (but not STIM1L) is increased in muscles from aged trained compared to aged mice, even if only the STIM1S resulted statistically different.

## Discussion

### Main Findings of the Study

In the present work, we showed that (i) STIM1 and ORAI1 are accumulated in TAs in muscle of aged mice (Figure 1); (ii) TAs, absent in fibers from adult mice while abundant in those from aged mice, are reduced in incidence and size following 15 months of voluntary running (Figure 3); (iii) the presence of TAs in EDL of aged mice correlates to a reduced ability to maintain contractile force during repetitive high-frequency stimulation, likely as the result of the lowered ability to refill internal Ca^2+^ stores *via* SOCE (Figures 4, 5); and (iv) voluntary exercise improves the fatigue resistance during repetitive stimulation (Figures 4, 5) and promotes the maintenance of SR and TT elements needed for the assembly of CEUs (Figure 6). Overall, our results suggest that (a) aging causes improper accumulation of STIM1 and ORAI1 in TAs and defective SOCE; and (b) long-term regular exercise strikingly reduces formation of TAs during aging, while promoting the maintenance of CEUs and improving the use of external Ca^2+^ during repetitive high-frequency stimulation.

### SOCE Is Dysfunctional in Muscles Containing TAs

TAs in aging mice stain positively for both STIM1 and ORAI1. These results agree with the work of Endo and colleagues, showing that TAs of patients with TAM are immunopositive for both proteins (Endo et al., 2015). The staining pattern for STIM1 appears to be quite dense and uniform, whereas the staining of ORAI1 is not as uniform.

(1) We have previously shown (Boncompagni et al., 2012) that tubes of TAs appear linked together by many small bridges (Boncompagni et al., 2012). Although the molecular nature of these electron-dense strands remains unknown, similar linkers are present between SR vesicles in fibers from control mice and between SR stack cisternae formed in muscle from mice subjected to acute exercise, both localized within the I band of the sarcomere [Figure 2; see also Boncompagni et al. (2017)]. Furthermore, electron-dense strands with similar length were reported between stacks of endoplasmic reticulum in HEK93 cells overexpressing STIM1 (Orci et al., 2009; Perni et al., 2015). Considering that STIM1 is localized throughout the I band in muscle fibers of control mice, we speculate that these little bridges could represent indeed aggregated STIM1 proteins, a hypothesis supported by immunogold staining in our previous studies (Boncompagni et al., 2017).
(2) ORAI1 is a Ca^2+^ release–activated Ca^2+^ channel of external membranes (Feske et al., 2006); hence, to be functional, it has to be localized either in the plasma membrane or in TTs. Our results show that ORAI1 also accumulated within TAs in muscles from aged mice. As TTs are mainly excluded from TAs [Figure 1; see also Boncompagni et al. (2012) for additional detail], and as Ca^2+^ entry is dysfunctional in aged muscles, it is plausible to argue that ORAI1 might be trapped in the SR tubes of TAs without being able to reach its functional destination in TTs. In line with this hypothesis, Thornton and colleagues showed that SOCE is significantly impaired in aged skeletal muscle, although the transcription levels of both STIM1 and ORAI1 were unchanged compared to young muscle (Thornton et al., 2011). Reduced SOCE activity could lead to impaired SR Ca^2+^ refilling upon repetitive contraction–relaxation cycles, and in turn, reduced availability of Ca^2+^ within the SR would underlie the lower ability to generate specific force during prolonged contraction (Zhao et al., 2008; Thornton et al., 2011; Michelucci et al., 2019). Consistent with these findings, we found that aged EDL muscles display a higher susceptibility to fatigue than adult muscles, during repetitive stimulation in presence of external Ca^2+^ (Figure 4). The lack of a further reduction of force in absence of extracellular Ca^2+^ influx in aged muscles (Figure 5) suggests that SOCE may be dysfunctional, possibly due to the accumulation of part of STIM1 and ORAI1 within TAs.

### Exercise Prevents TAs Formation and Preserves Structural Elements of CEUs

We have recently provided evidence that exercise drives the formation of CEUs, new intracellular junctions representing sites of functional and dynamic STIM1-ORAI1 association that promote Ca^2+^ entry and limit muscle fatigue during repetitive stimulation (Boncompagni et al., 2017; Michelucci et al., 2019). While the formation of CEUs seems to be important for the optimal coupling between STIM1 and ORAI1, the accrual of TAs might have the opposite effect of sequestering part of the two proteins in a way that they are no longer able to interact properly.

In this study, we found that regular exercise in wheel cages significantly reduces the accrual of TAs during aging and promotes the maintenance of the structural elements required for the assembly of functional CEUs, i.e., SR stacks and T tubules at the I band (Figure 6). Importantly, the exercise-induced maintenance of CEU elements is accompanied by recovered capability of EDL muscles to use external Ca^2+^ (Figures 4, 5), suggesting that STIM1 and ORAI1 in aged trained mice are better available for functional activation of SOCE than in aged sedentary mice. Our results are supported by a recent article from Fodor and colleagues, showing that skeletal muscle Ca^2+^ homeostasis and force significantly improved in aged mice following voluntary wheel training (Fodor et al., 2020).

Stiber and colleagues proposed two functionally distinct pools of STIM1, one at the triad and another one within the lSR at the I band (Stiber et al., 2008). Recently, Darbellay and collegues provided a possible molecular explanation for these two STIM1 pools. Indeed, the authors identified a STIM1 splice variant highly expressed in skeletal muscle (STIM1L) proposed to be preassembled with ORAI1 at the triad to mediate rapid SOCE, while graded recruitment of additional SOCE during prolonged activity could be mediated by STIM1S (Darbellay et al., 2009, 2011; Michelucci et al., 2018). Being STIM1S distributed throughout the lSR at the I band, this isoform would indeed be in the right position to contribute to the assembly of CEUs during exercise. This idea is supported by our data showing a correlation between increased expression of STIM1S and maintenance of CEU elements in trained aged mice.

## Conclusion

For a long time, the physiological role of extracellular Ca^2+^ in skeletal muscle function has been overlooked, as not required for mechanical EC coupling (Schneider and Chandler, 1973; Rios et al., 1991). Over the past two decades, though, the importance of SOCE in maintaining proper contractile function during prolonged activity has been taken into account (Pan et al., 2002, 2014; Putney, 2011b; Boncompagni et al., 2017; Michelucci et al., 2019). A deeper understanding of the molecular mechanisms that promote functional (and dysfunctional) interaction between STIM1 and ORAI1 in SOCE would be crucial for the development of safe/effective therapeutic interventions to limit susceptibility to fatigue and weakness in aging and in those muscle diseases caused by altered Ca^2+^ homeostasis. The results of the present work suggest that TAs could represent intracellular bins for dysfunctional accumulation of proteins, including STIM1 and ORAI1. The presence of TAs is accompanied by impaired ability to restore internal Ca^2+^ stores from extracellular space, which could contribute to both muscle weakness and increased susceptibility to fatigue during aging. Our hypothesis agrees with the work by Thornton and colleagues suggesting that SOCE contributes to normal contractility in young, but not in aged skeletal muscle (Thornton et al., 2011), although it is difficult to determine whether TAs form as a consequence of an altered Ca^2+^ handling, or else if they are the cause of muscle dysfunction. Undoubtedly, our experiments indicate that long-term regular exercise counteracts the formation of TAs and promotes the assembly of functional CEUs, which is accompanied by an improved capability of fibers to use extracellular Ca^2+^.

## Data Availability Statement

The original contributions presented in the study are included in the article/Supplementary Material, further inquiries can be directed to the corresponding author/s.

## Ethics Statement

The animal study was reviewed and approved by National Committee for the protection of animals used for scientific purposes (D. lgs n.26/2014), Italian Ministry of Health Viale Giorgio Ribotta, 5 – 00144 – Roma.

## Author Contributions

SB and FP conceived and directed the study. SB, CP, AM, and LP performed the experimental work and data analysis. In detail, CP, LP, and SB performed experiments and data analysis of Figures 1–3, 6 and Supplementary Figures 1, 2. CP also performed the Western blot analysis (Supplementary Figure 3). AM performed functional experiments and analysis of Figures 4, 5. Finally, SB, AM, and FP wrote and edited the manuscript. All authors contributed to the article and approved the submitted version.

## Conflict of Interest

The authors declare that the research was conducted in the absence of any commercial or financial relationships that could be construed as a potential conflict of interest.

## Abbreviations

Ca^2+^: calcium
CEU: Ca^2+^ entry unit
CM: confocal microscopy
CRU: Ca^2+^ release unit
EDL: extensor digitorum longus
EC coupling: excitation-contraction coupling
EM: electron microscopy
RYR1: ryanodine receptor type 1
SERCA: sarco/endoplasmic reticulum ATPase
SOCE: store-operated Ca^2+^ entry
SR: sarcoplasmic reticulum
STIM1: stromal interaction molecule-1
TA: tubular aggregate
TAM: TA myopathy
TT: transverse tubule
WT: wild type.
