# Supplementary material for: Long-Term Exercise Reduces Formation of Tubular Aggregates and Promotes Maintenance of Ca2+ Entry Units in Aged Muscle
Source: Front Physiol. 2021 Jan 5;11:601057. doi: 10.3389/fphys.2020.601057 (PMC7813885; doi:10.3389/fphys.2020.601057)
Supplement: Supplementary file 1 [file Data_Sheet_1.pdf]

Supplemental Figures and Tables

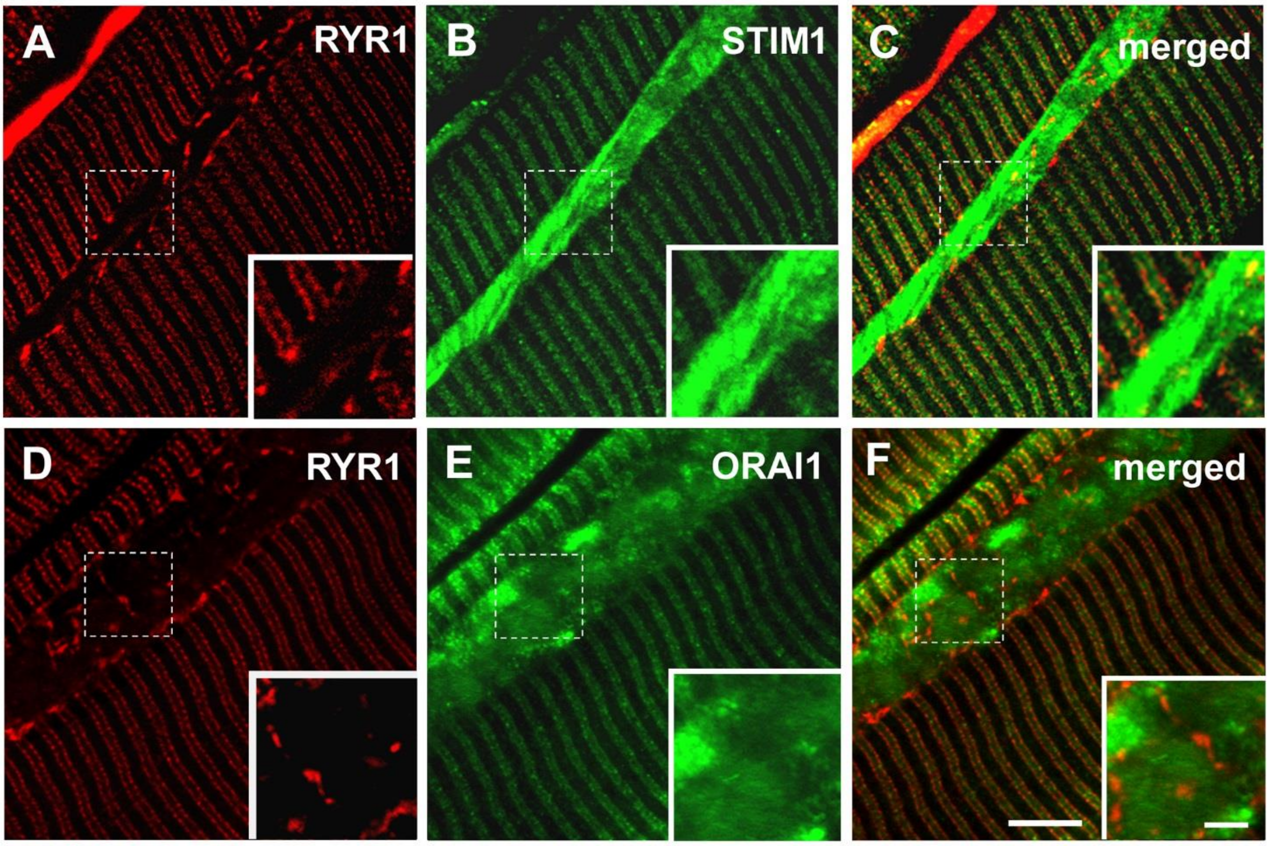

Figure S1. Raw images for the individual fluorescence channel used to construct the overlay images in Figure 1. In these experiments EDL fibers from aged mice were double-labeled for RYR1 vs. STIM1 (A-C) and RYR1 vs. ORAI1 (D-F). *Scale bar: A-F, 5 µm (insets 2.5 µm).*

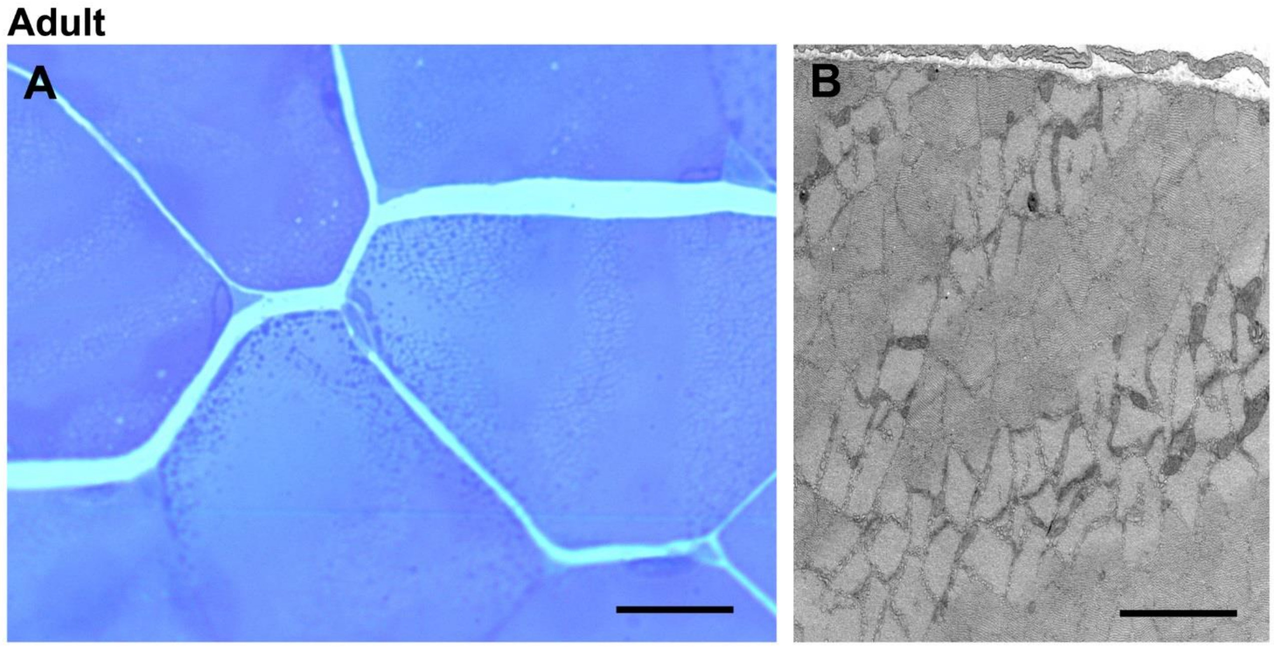

Figure S2. Representative histological and EM images of adult EDL fibers from mice. Tubular aggregates are never found in EDL muscle from adult mice. *Scale bars: A, 10 μm; B, 2μm.*

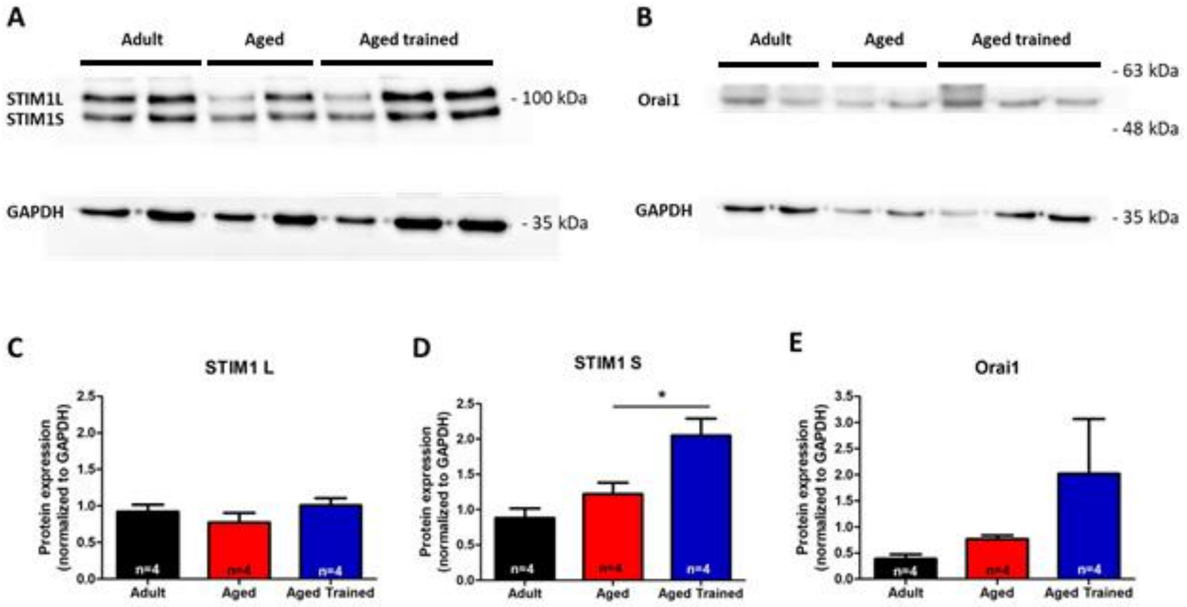

Figure S3. Evaluation of STIM1 and Orai1 expression by WB in EDL muscle homogenates. A-B) Representative immuno-blots of EDL muscle homogenates from adult, aged control, and aged-trained mice showing expression levels of STIM1 splice variants (long: STIM1L; short: STIM1S) and of ORAI1. GAPDH was used as a loading control. C-E) Bar blots showing the quantitative analysis of STIM1 and ORAI1 relative expression, normalized to GAPDH. *Data are shown as mean ± SEM; *p<0.05; n = number of EDL muscles.*

Table S1. Quantitative analyses of percentage of EDL fibers containing TAs (column A), the number of TAs per fiber (column B), and the average size of TAs (column C) in EDL muscles from adult, aged control, and aged trained mice. Data contained in this Table were used to construct the bar graphs in Fig. 3 E - G.

	**A**<br>**% of fibers with TAs**	**B**<br>**n. of TAs / fibers**	**C**<br>**Average size of TAs (μm^2)**
**Adult**	0.0 ± 0.0	0.0 ± 0.0	0.0 ± 0.0
**Aged**	50.6 ± 9.7 (885)	5.8 ± 0.5 (298)	21.2 ± 4.4 (303)
**Aged trained**	7.7 ± 1.2** (1258)	2.9 ± 0.9**(885)	17.0 ± 0.9* (212)

*Data are shown as mean ± SEM ; **p<0.01 in columns A and B vs. aged; *p<0.05 in column C vs. aged; Samples size: 3 mice for each group. In parenthesis: n. of fibers analyzed.*

Table S2. Quantitative EM analyses of the incidence of SR stacks and TT length ~~extension~~ at the I band. Data contained in this Table were used to construct the bar graphs in Fig. 6 G and H.

	**A**<br>**n. of stacks /100 μm^2**	**B**<br>**TT length (μm) /100 μm^2**
**Adult**	1.6 ± 0.3 (61)	2.4 ± 0.8 (12)
**Aged**	1.8 ± 0.3 (50)	0.6 ± 0.2* (19)
**Aged trained**	3.6 ± 0.6* (40)	1.9 ± 0.4* (22)

*Data are shown as mean ± SEM;*p < 0.01 vs. aged and adult in panel G. Samples size: 3 mice for each group. In parenthesis: n. of fibers analyzed.*
